# Mirror therapy combined with defecation motor imagery and repetitive transcranial magnetic stimulation improves post-stroke constipation: a retrospective study with EEG biomarker analysis

**DOI:** 10.3389/fneur.2026.1818303

**Published:** 2026-07-10

**Authors:** Jiansong Yu, Jingyuan Lin

**Affiliations:** 1 Rehabilitation Medicine Department, Taizhou Hospital of Zhejiang Province Affiliated to Wenzhou Medical University, Taizhou, Zhejiang, China; 2 Rehabilitation Medicine Department, Fujian Provincial Geriatric Hospital, Fuzhou, China

**Keywords:** constipation, frontal lobe infarction, mirror therapy, motor imagery, transcranial magnetic stimulation (TMS)

## Abstract

**Background:**

Post-stroke constipation is prevalent after frontal lobe infarction and may reflect disrupted cortical–autonomic regulation. Neurorehabilitation strategies targeting cortical plasticity may offer therapeutic benefit.

**Objective:**

To evaluate whether mirror therapy combined with defecation motor imagery and repetitive transcranial magnetic stimulation (rTMS) improves bowel function, and to explore associated cortical changes using EEG-derived event-related desynchronization (ERD).

**Methods:**

In this single-center retrospective study, 98 patients with frontal lobe infarction and Rome IV-defined constipation were analyzed. Forty-eight received mirror therapy + motor imagery + rTMS in addition to routine rehabilitation, and 50 received routine care alone. Primary outcomes included Wexner Constipation Score, weekly bowel frequency, and PAC-QOL. EEG μ/β-band ERD during defecation motor imagery was quantified. Between-group differences and correlations were analyzed.

**Results:**

After 8 weeks, the intervention group showed significantly greater improvement in Wexner score, bowel frequency, and PAC-QOL compared with controls (all *P* < 0.01; Cohen's d 0.60–0.82). Enhanced μ/β ERD was observed only in the intervention group. ERD changes correlated moderately with Wexner improvement (*r* = 0.56).

**Conclusions:**

Combined mirror therapy, motor imagery, and rTMS were associated with improved bowel function and increased task-related cortical activation. EEG-derived ERD may serve as a candidate biomarker for bowel-focused neurorehabilitation.

## Introduction

Constipation is one of the most prevalent but least addressed complications after stroke, especially in patients with frontal lobe infarction, and may involve disrupted cortical regulation of visceral function. Beyond constipation itself, post-stroke gastrointestinal dysfunctions may include impaired gut motility, altered autonomic regulation, and increased susceptibility to gastrointestinal infections, all of which can negatively influence rehabilitation participation and overall recovery. Recent studies have further highlighted the clinical burden of gastrointestinal complications, including Clostridium difficile infection, in stroke patients, with significant implications for hospitalization duration, healthcare costs, and functional outcomes (1). These findings support the importance of targeting bowel dysfunction as part of comprehensive neurorehabilitation rather than considering it merely a secondary symptom. Epidemiological studies estimate that up to half of stroke survivors experience varying degrees of bowel dysfunction, ranging from delayed colonic transit to frank constipation (2), which may reflect both peripheral and central (cortical) contributions to bowel control. Persistent constipation reduces quality of life, increases caregiver burden, and has been linked with poorer participation in rehabilitation programs and delayed neurological recovery, highlighting the need to consider central neural mechanisms in rehabilitation strategies.

Beyond mechanical issues, the “brain–gut axis” model has attracted growing attention. Cortical injury, particularly in regions such as the prefrontal cortex, insula, and cingulate gyrus, may disrupt autonomic and visceral regulation pathways, including hypothalamic interactions, thereby aggravating bowel dysfunction and establishing a vicious cycle that interferes with global recovery (3). Prior neuroimaging studies have linked cortical and subcortical activations with bowel-related motor and sensory control. Functional MRI studies of anorectal sensation, rectal distension, and pelvic floor contraction have demonstrated activation of the supplementary motor area, medial sensorimotor cortex, insula, anterior cingulate cortex, and prefrontal regions (4–7). These findings suggest that defecation-related sensory and motor control depends on a distributed cortical–subcortical network rather than peripheral mechanisms alone, thereby providing a mechanistic rationale for interventions targeting these networks. However, MRI/fMRI was not used in the present study because this was a retrospective clinical rehabilitation study, and functional neuroimaging was not routinely included in the assessment pathway for post-stroke constipation in our center. In addition, repeated fMRI assessment during defecation imagery would have increased examination cost, patient burden, and implementation difficulty in older post-stroke patients. Therefore, EEG-derived μ/β event-related desynchronization was selected as a more feasible, repeatable, and rehabilitation-compatible marker of cortical engagement during defecation motor imagery. In addition, repeated fMRI assessment during defecation imagery would have increased examination cost, patient burden, and implementation difficulty in older post-stroke patients. Therefore, EEG-derived μ/β event-related desynchronization was selected as a more feasible, repeatable, and rehabilitation-compatible marker of cortical engagement during defecation motor imagery. Despite this burden, stroke rehabilitation research continues to prioritize motor and language recovery, leaving bowel-related dysfunction underexplored and under-treated (8).

In parallel with this neglect, the last decade has seen significant advances in neurorehabilitation strategies. Mirror therapy has emerged as a low-cost, easily implemented technique that exploits visual feedback to induce motor learning. Systematic reviews and meta-analyses confirm its effectiveness in improving upper limb function and daily activities in chronic stroke (9, 10). More recently, virtual reality–enhanced mirror therapy has been tested, with randomized controlled trials showing additional gains in satisfaction and functional performance (11). These approaches are complemented by motor imagery, which recruits overlapping neural substrates with actual motor execution and modulates cortical excitability (12). Although mirror therapy and motor imagery are well established in limb motor rehabilitation, their application to visceral or pelvic floor motor control remains limited. Preliminary studies suggest that motor imagery can engage cortical networks involved in pelvic floor and autonomic control, indicating potential physiological relevance for bowel-related rehabilitation, yet systematic investigation in post-stroke constipation is lacking.

Neurophysiological monitoring provides an essential mechanistic window into these interventions. Electroencephalography (EEG) is particularly well suited for rehabilitation research due to its high temporal resolution, portability, and sensitivity to task-specific oscillatory changes. Event-related desynchronization (ERD) in the mu (8–13 Hz) and beta (14–25 Hz) bands reflects cortical activation during both executed and imagined movements (13, 14). In stroke populations, the degree of mu/beta ERD has been associated with motor impairment severity and recovery potential (15). Recent scoping reviews and experimental studies have highlighted EEG not only as a tool for monitoring but also as a potential biomarker for predicting rehabilitation response (16). Importantly, these measures may also indirectly reflect cortical engagement during bowel-related motor control, providing a mechanistic link to visceral function recovery.

Alongside behavioral training, neuromodulation techniques such as repetitive transcranial magnetic stimulation (rTMS) have been increasingly adopted in stroke rehabilitation. Meta-analyses show that rTMS applied over motor areas can enhance upper limb motor recovery and improve daily function, with effect sizes that justify its growing inclusion in guidelines (17). Beyond motor rehabilitation, rTMS has been explored in cognitive and affective disorders, underscoring its capacity to modulate cortical networks beyond the motor cortex. Nevertheless, almost no work has integrated rTMS into protocols targeting bowel function, despite the plausible role of frontal regions in autonomic regulation and the potential for neuromodulation to enhance plasticity in circuits relevant to defecation control (18).

Taken together, these developments underscore a striking gap. Stroke-related constipation is common, burdensome, and mechanistically linked to cortical dysfunction, yet systematic trials are lacking. Interventions with proven efficacy in limb motor domains—mirror therapy, motor imagery, and rTMS—remain untested in visceral domains. EEG offers validated neurophysiological markers of cortical reorganization, but their relevance to bowel function recovery is unknown. The present retrospective study is designed to bridge this gap. Specifically, it evaluates whether mirror therapy combined with defecation motor imagery and adjunctive TMS improves bowel outcomes—including Wexner scores, bowel frequency, and quality of life—in patients with frontal lobe infarction, and whether EEG ERD in mu/beta bands provides mechanistic evidence of cortical engagement and correlates with clinical gains. By integrating rehabilitation techniques with neuromodulation and EEG biomarkers, this study aims to advance post-stroke constipation management from an overlooked complication toward a data-driven, mechanistically informed, and clinically actionable target.

## Materials and methods

This study was designed as a retrospective analysis of clinical and electrophysiological data from patients with frontal lobe infarction complicated by constipation who were admitted to Fujian Provincial Geriatric Hospital between January 2020 and December 2024. The protocol was reviewed and approved by the Ethics Committee of Fujian Provincial Geriatric Hospital (approval number: 20260504). All procedures adhered to the principles of the Declaration of Helsinki.

### Patient selection and diagnostic criteria

Eligible participants were adults aged 40–85 years with imaging-confirmed frontal lobe infarction and a clinical diagnosis of post-stroke constipation. Stroke lesions were confirmed by MRI and restricted to the frontal lobe based on radiological reports. Lesion laterality (left, right, or bilateral), cortical vs. subcortical involvement, and approximate lesion extent were extracted from imaging records. Patients with extensive multi-lobar infarctions were excluded to reduce anatomical heterogeneity. Time since stroke onset at enrollment was recorded and categorized as subacute (1–6 months) or chronic (>6 months). Stroke diagnosis was based on World Health Organization criteria, supported by CT or MRI confirmation of infarction location (19). Constipation was diagnosed according to the Rome IV criteria, which define functional constipation by the presence of at least two symptoms including straining, hard stools, sensation of incomplete evacuation, anorectal obstruction, manual maneuvers, or fewer than three spontaneous bowel movements per week (20). Patients with pre-stroke chronic gastrointestinal disease, severe cognitive impairment (Mini-Mental State Examination < 10), or contraindications to TMS or EEG were excluded.

During the study period (January 2020 to December 2024), a total of 126 patients with imaging-confirmed frontal lobe infarction were screened for eligibility. Of these, 98 met the inclusion criteria and were included in the final analysis. Twenty-eight patients were excluded for the following reasons: pre-existing chronic gastrointestinal disease (*n* = 2), severe cognitive impairment (MMSE < 10) (*n* = 11), contraindications to TMS or EEG (*n* = 6), multi-lobar or non-frontal infarction (*n* = 3), or incomplete clinical data (*n* = 6).

### Intervention protocol

Patients were divided into two groups: (1) intervention group, which received mirror therapy combined with defecation motor imagery and adjunctive repetitive transcranial magnetic stimulation (rTMS), in addition to routine rehabilitation; and (2) control group, which received routine rehabilitation only. Group allocation was based on treatment decisions made jointly by attending physicians and patient preference, considering factors such as age, sex, severity of stroke, baseline bowel function, and cognitive status. As this was a retrospective observational study rather than a randomized controlled trial, the allocation was not randomized, but these criteria were applied to minimize systematic differences between groups.

#### Mirror therapy and motor imagery

Participants were seated comfortably in an upright position. A vertical mirror (approximately 40 × 60cm) was placed in the sagittal plane in front of the abdomen and pelvis at a distance of approximately 50–70 cm, oriented to reflect the lower abdominal wall and pelvic region. Each session began with 15 min of mirror therapy, during which patients observed their abdominal movements while performing slow, voluntary abdominal expansion and relaxation. They were guided to imagine that the mirrored image represented internal bodily movement associated with defecation. Verbal instructions included: “Imagine that the movement you see in the mirror corresponds to your internal bowel movement,” and “Focus on the sensation of coordinated abdominal and pelvic relaxation.”

This was followed by 20 min of guided defecation motor imagery, divided into four 5-min blocks interspersed with 1-min rest periods. The intervention aimed to enhance visuomotor integration and promote embodiment of the observed abdominal movement. Although no formal embodiment scale was administered, patients were encouraged to experience the reflection as representing their own bodily action.

#### TMS stimulation

High-frequency rTMS (10 Hz) was delivered using a Magstim Rapid^2^ stimulator with a figure-of-eight coil targeting the bilateral medial frontal cortex (Fz electrode site according to the international 10–20 EEG system) (17), approximating the medial prefrontal and supplementary motor area (SMA) regions implicated in pelvic floor and visceral motor control. rTMS was applied for 10 min at the end of each session.

Each participant received 3 sessions per week for 8 weeks. The intervention protocol was structured to clearly specify session duration, repetitions, imagery timing, and rest periods to enhance reproducibility.

### Routine rehabilitation

All participants received standard post-stroke rehabilitation and constipation nursing management during the 8-week period. Standard rehabilitation consisted of (1) conventional physiotherapy focusing on mobility and trunk control, (2) occupational therapy targeting basic activities of daily living, and (3) standardized nursing education for bowel management.

#### Schedule and intensity

Physiotherapy and occupational therapy were delivered 5 days/week, with a total of approximately 60 min/day (physiotherapy: 30 min; occupational therapy: 30 min), for 8 weeks. Standard bowel management included scheduled toileting attempts (e.g., after breakfast), hydration and dietary guidance, abdominal massage as needed, and review of constipation-related medications.

To minimize attention bias, the control group received equal therapist contact time as the intervention group. Specifically, an additional 45-min session matched in frequency and duration was provided, consisting of non-specific health education and relaxation training without motor imagery, mirror feedback, or rTMS.

### Outcome measures

Clinical and neurophysiological outcomes were assessed at baseline and after 8 weeks of intervention.

Constipation severity: Evaluated with the Wexner Constipation Score (range 0–30, higher scores indicate more severe constipation).

Bowel movement frequency: Number of spontaneous bowel movements per week recorded from patient diaries.

Quality of life: Patient Assessment of Constipation Quality of Life (PAC-QOL), with lower scores representing better quality of life.

Cognitive function and stroke severity: Baseline Mini-Mental State Examination (MMSE) and National Institutes of Health Stroke Scale (NIHSS) scores were collected for comparability.

EEG recordings: EEG was acquired using a 64-channel Brain Products system (BrainVision, Germany) at 1,000 Hz sampling. During defecation motor imagery tasks, ERD in mu (8–13 Hz) and beta (14–25 Hz) bands was calculated using event-related spectral perturbation methods, averaged across sensorimotor channels (Cz, FCz, CPz). EEG data were band-pass filtered (1–40 Hz), and trials contaminated by eye or muscle artifacts were rejected using semi-automatic procedures. ERD was calculated relative to a pre-imagery baseline period (−1,000 to 0 ms). ERD analysis focused on midline sensorimotor electrodes (FCz, Cz, and CPz) based on prior evidence that pelvic floor contraction and ano-rectal sensory processing engage medial primary motor cortex and supplementary motor area (SMA) (18). Neuroimaging studies have demonstrated midline cortical activation during pelvic floor and rectal stimulation tasks. Given the somatotopic representation of trunk and pelvic musculature along the medial sensorimotor cortex, midline electrodes were selected to capture task-related cortical desynchronization. Electrode impedances were maintained below 10 kΩ before each recording session. EEG signals were initially referenced to FCz during acquisition and subsequently re-referenced to the common average during offline preprocessing. Continuous EEG data were segmented into epochs corresponding to individual defecation motor imagery trials. Spectral features and ERD measures were computed for each epoch relative to a pre-imagery baseline period (−1,000 to 0 ms) and then averaged across trials for statistical analysis.

Safety monitoring: Adverse events related to TMS or mirror therapy were recorded.

### Statistical analysis

Continuous variables were expressed as mean ± standard deviation, while categorical variables were expressed as frequencies and percentages. Group differences were analyzed using independent *t*-tests or chi-square tests where appropriate. Within-group changes were assessed by paired *t*-tests. Pearson correlation analysis was performed to examine associations between EEG ERD changes and improvements in Wexner score, bowel frequency, and PAC-QOL. A two-tailed *P* < 0.05 was considered statistically significant. Analyses were performed with SPSS version 27.0 (IBM, USA). No formal sample size calculation was performed because of the retrospective nature of the study. All eligible patients meeting the inclusion criteria during the study period were included in the analysis.

Normality was assessed using the Shapiro–Wilk test. Sensitivity analyses adjusting for age and baseline NIHSS yielded consistent results.

## Result

### Baseline characteristics and clinical outcomes

As shown in Table 1, Baseline characteristics were generally well balanced between the intervention and control groups, with no significant differences in demographics, stroke-related variables, comorbidities, or constipation-related factors (all *P* > 0.05). After intervention, the mirror therapy + motor imagery + TMS group showed significantly greater improvements in bowel function compared with the control group, as evidenced by lower Wexner constipation scores, increased bowel movement frequency, and improved PAC-QOL scores (all *P* < 0.01).

**Table 1:** Baseline demographic and clinical characteristics of patients with frontal lobe infarction in the intervention and control groups.

Variable	Intervention	Control	t/χ^2^	*P* value
Age	64.05 ± 7.34	63.52 ± 6.87	0.37	0.71
BMI	24.14 ± 2.51	23.97 ± 3.02	0.29	0.77
Time since stroke (days)	74.12 ± 27.83	64.44 ± 30.19	1.65	0.10
Infarct volume (mL)	23.23 ± 6.87	21.46 ± 8.36	1.15	0.25
NIHSS	7.94 ± 2.69	7.84 ± 2.51	0.19	0.85
mRS	2.92 ± 0.90	2.76 ± 0.74	0.94	0.35
Barthel	64.00 ± 15.00	69.26 ± 14.23	−1.78	0.08
MMSE	25.60 ± 2.38	25.86 ± 2.19	−0.55	0.58
MoCA	23.48 ± 3.54	22.86 ± 3.16	0.91	0.36
HADS_D	6.40 ± 3.19	6.00 ± 3.11	0.62	0.54
PSQI	8.29 ± 3.44	7.86 ± 3.00	0.66	0.51
Constipation duration (months)	9.69 ± 5.83	10.76 ± 6.29	−0.88	0.38
Fiber intake (g/day)	14.73 ± 4.48	17.14 ± 5.84	−2.3	0.02
Fluid intake (mL/day)	1,401.73 ± 332.87	1,361.96 ± 332.44	0.59	0.56
TSH (μIU/mL)	1.86 ± 0.82	1.97 ± 0.78	−0.67	0.50
Calcium (mmol/L)	2.29 ± 0.06	2.30 ± 0.08	−0.57	0.57
Hemoglobin (g/dL)	13.18 ± 1.02	13.06 ± 1.47	0.48	0.63
Albumin (g/L)	40.27 ± 3.85	39.28 ± 4.10	1.23	0.22
Baseline Wexner score	14.51 ± 3.22	13.88 ± 2.63	1.07	0.29
Baseline bowel frequency	2.48 ± 0.88	2.24 ± 0.93	1.3	0.20
Baseline PAC-QOL score	2.86 ± 0.50	2.87 ± 0.53	−0.06	0.95
Sex	F: 20 (42%); M: 28 (58%)	F: 21 (42%); M: 29 (58%)	0	1.00
LesionSide	Left: 27 (56%); Right: 21 (44%)	Left: 29 (58%); Right: 21 (42%)	0	1.00
Dysphagia	No: 34 (71%); Yes: 14 (29%)	No: 38 (76%); Yes: 12 (24%)	0.12	0.73
Independent gait	No: 35 (73%); Yes: 13 (27%)	No: 29 (58%); Yes: 21 (42%)	1.79	0.18
Hypertension	No: 19 (40%); Yes: 29 (60%)	No: 25 (50%); Yes: 25 (50%)	0.69	0.40
Diabetes	No: 33 (69%); Yes: 15 (31%)	No: 36 (72%); Yes: 14 (28%)	0.02	0.90
Hyperlipidemia	No: 30 (62%); Yes: 18 (38%)	No: 24 (48%); Yes: 26 (52%)	1.54	0.22
Atrial fibrillation	No: 40 (83%); Yes: 8 (17%)	No: 47 (94%); Yes: 3 (6%)	1.83	0.18
Opioid use	No: 38 (79%); Yes: 10 (21%)	No: 36 (72%); Yes: 14 (28%)	0.35	0.56
Anticholinergic use	No: 40 (83%); Yes: 8 (17%)	No: 40 (80%); Yes: 10 (20%)	0.03	0.87
Antidepressant use	No: 35 (73%); Yes: 13 (27%)	No: 36 (72%); Yes: 14 (28%)	0	1.00
CCB use	No:38 (79%); Yes:10 (21%)	No:43 (86%); Yes:7 (14%)	0.39	0.53
RomeIV_constipation	No: 4 (8%); Yes: 44 (92%)	No: 3 (6%); Yes: 47 (94%)	0	0.96
Bristol type	1: 9 (19%); 2: 15 (31%); 3: 17 (35%); 4: 7 (15%)	1: 9 (18%); 2: 23 (46%); 3: 17 (34%); 4: 1 (2%)	6.15	0.10
Prior laxative use	No: 24 (50%); Yes: 24 (50%)	No: 25 (50%); Yes: 25 (50%)	0	1.00
Wexner baseline	14.51 ± 3.22	13.88 ± 2.63	1.07	0.29
Wexner post	8.35 ± 3.59	11.84 ± 2.76	−5.39	< 0.01
Wexner Δ change	6.17 ± 1.41	2.03 ± 1.11	16.06	< 0.01
Bowel frequency (/week) baseline	2.48 ± 0.88	2.24 ± 0.93	1.3	0.20
Bowel frequency (/week) post	4.38 ± 1.22	3.10 ± 1.11	5.44	< 0.01
Bowel frequency (/week) Δ change	1.90 ± 0.76	0.86 ± 0.55	7.81	< 0.01
PAC-QOL baseline	2.86 ± 0.50	2.87 ± 0.53	−0.06	0.95
PAC-QOL post	2.08 ± 0.58	2.53 ± 0.53	−4.07	< 0.01
PAC-QOL Δ change	0.78 ± 0.26	0.33 ± 0.23	9.04	< 0.01

*P* values are reported to three decimal places, and values smaller than 0.001 are presented as *P* < 0.001.

### Clinical outcomes after intervention

As shown in Table 2, both groups demonstrated significant within-group improvements in bowel function compared with baseline (all *P* < 0.05). However, between-group comparisons of Δ changes showed that the mirror therapy + motor imagery + TMS group had a larger reduction in Wexner constipation scores, a greater increase in weekly bowel movement frequency, and a more pronounced improvement in PAC-QOL scores than the control group. Therefore, the between-group difference was mainly reflected in the magnitude of improvement rather than in the presence or absence of improvement. The Δ-change comparisons were associated with medium-to-large effect sizes, indicating clinically meaningful between-group differences in treatment response.

**Table 2:** Within-group changes and between-group comparisons of bowel function and quality-of-life outcomes after 8 weeks of intervention.

Outcome	Group/Comparison	Baseline (Mean ±SD)	Post (Mean ±SD)	Δ Improvement (Mean ±SD)	*t* value	*P* value	Effect size (d)	95% CI
Wexner	Intervention	14.51 ± 3.22	8.35 ± 3.59	6.17 ± 1.41	30.28	< 0.001	—	—
Wexner	Control	13.88 ± 2.63	11.84 ± 2.76	2.03 ± 1.11	12.91	< 0.001	—	—
Wexner	Between-group Δ	—	—	4.13	16.06	< 0.001	3.27	2.58–3.96
Bowel frequency (/week)	Intervention	2.48 ± 0.88	4.38 ± 1.22	1.90 ± 0.76	−17.43	< 0.001	—	—
Bowel frequency (/week)	Control	2.24 ± 0.93	3.10 ± 1.11	0.86 ± 0.55	−11.05	< 0.001	—	—
Bowel frequency (/week)	Between-group Δ	—	—	1.04	7.81	< 0.001	1.57	1.11–2.03
PAC-QOL	Intervention	2.86 ± 0.50	2.08 ± 0.58	0.78 ± 0.26	20.61	< 0.001	—	—
PAC-QOL	Control	2.87 ± 0.53	2.53 ± 0.53	0.33 ± 0.23	10.48	< 0.001	—	—
PAC-QOL	Between-group Δ	—	—	0.45	9.04	< 0.001	1.84	1.38–2.29

### Clinical outcomes after intervention

**Figure 1:**
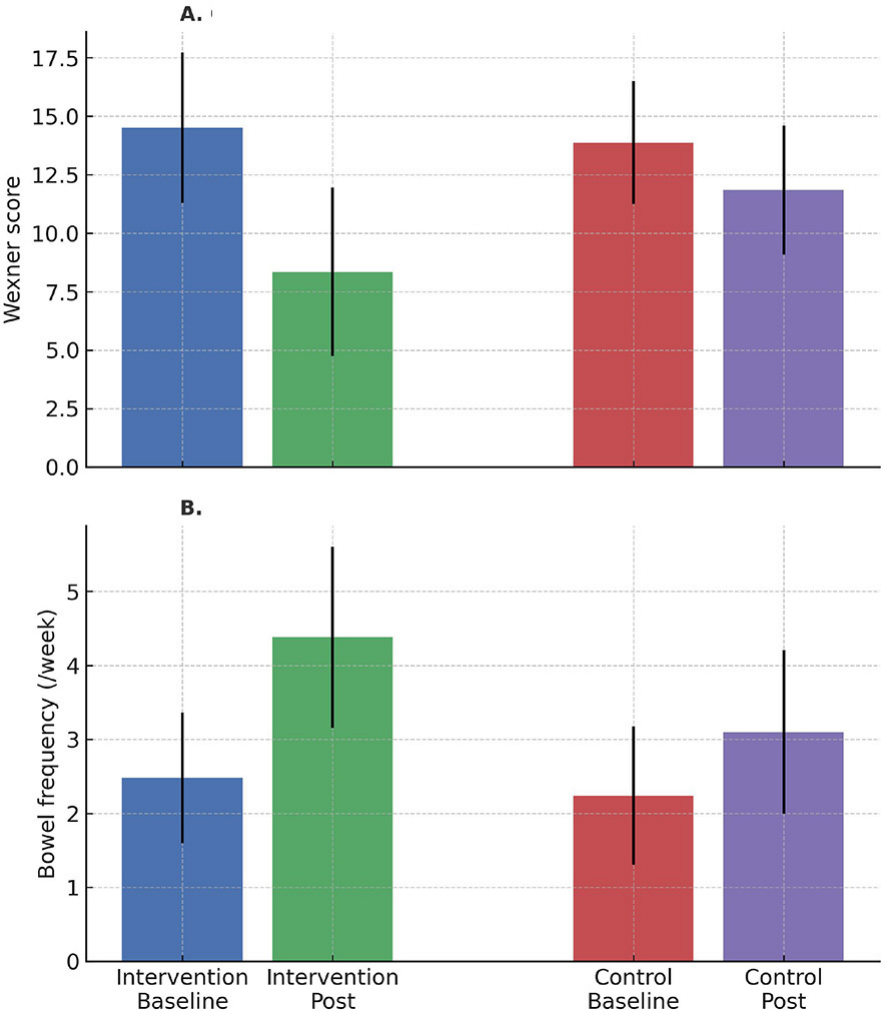
Changes in bowel function outcomes after 8 weeks of intervention. **(A)** Changes in Wexner constipation scores. **(B)** Changes in weekly bowel movement frequency. The symbol *** indicates statistical significance (*P* < 0.001).

As illustrated in Figure 1, both groups showed significant within-group improvements in bowel function after intervention. However, compared with the control group, the mirror therapy + motor imagery + TMS group demonstrated a significantly greater reduction in Wexner constipation scores (Figure 1A) and a greater increase in weekly bowel movement frequency (Figure 1B), indicating superior clinical efficacy.

### Neurophysiological changes after intervention

**Figure 2:**
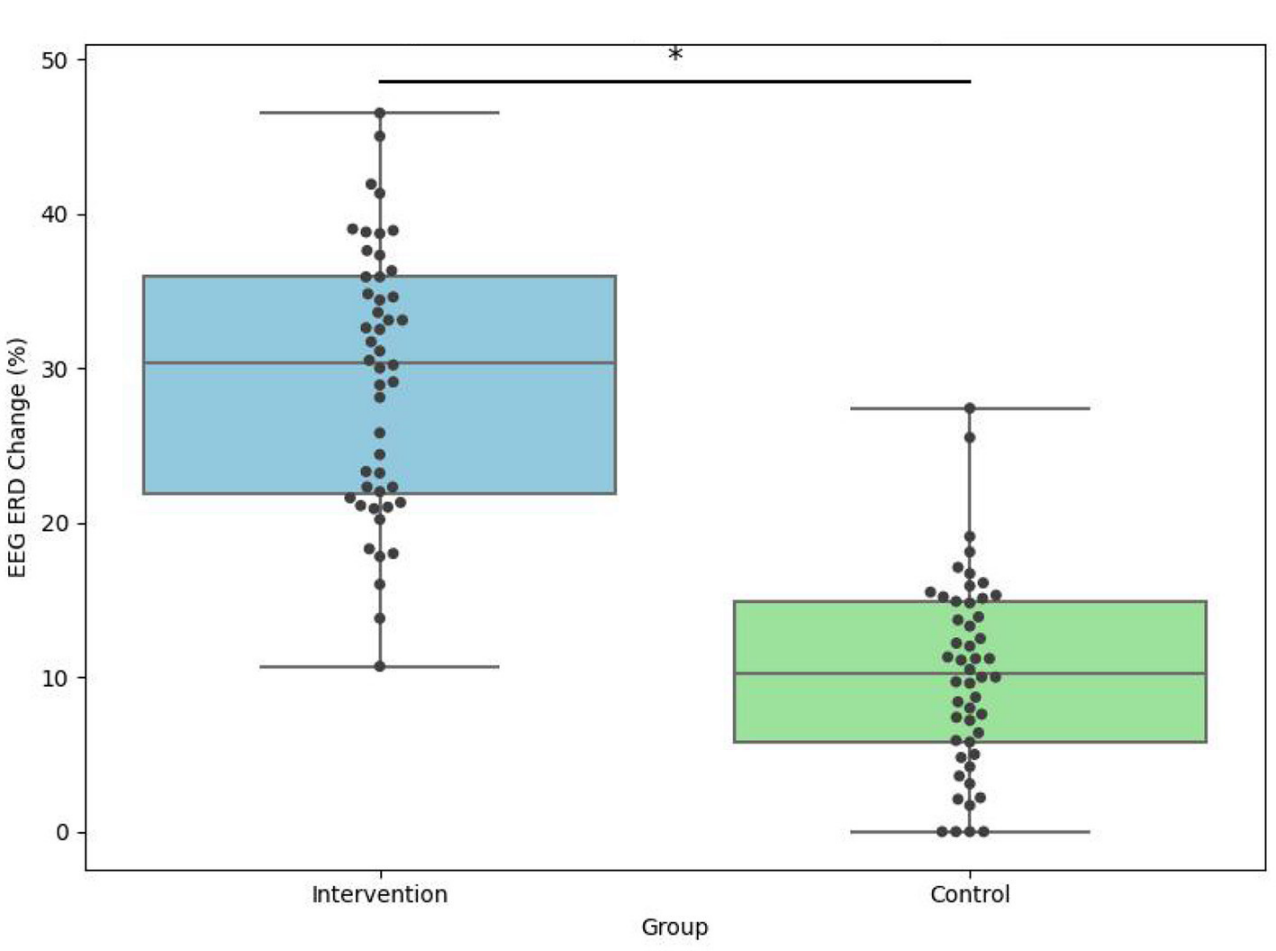
Boxplot comparison of EEG event-related desynchronization (ERD) changes during defecation motor imagery between the intervention and control groups. ERD comparison between groups. The symbol *indicates statistical significance (*P* < 0.05).

As shown in Figure 2, the intervention group demonstrated significantly greater EEG event-related desynchronization (ERD) changes during defecation motor imagery compared with the control group after treatment. ERD values, expressed as percentage power changes relative to baseline, were significantly enhanced in the intervention group (*P* < 0.05), whereas the control group showed only minimal changes. These findings suggest that mirror therapy combined with motor imagery and TMS may facilitate cortical activation and functional reorganization associated with bowel-related motor control.

### Correlation between EEG changes and clinical improvements in the overall cohort

**Figure 3:**
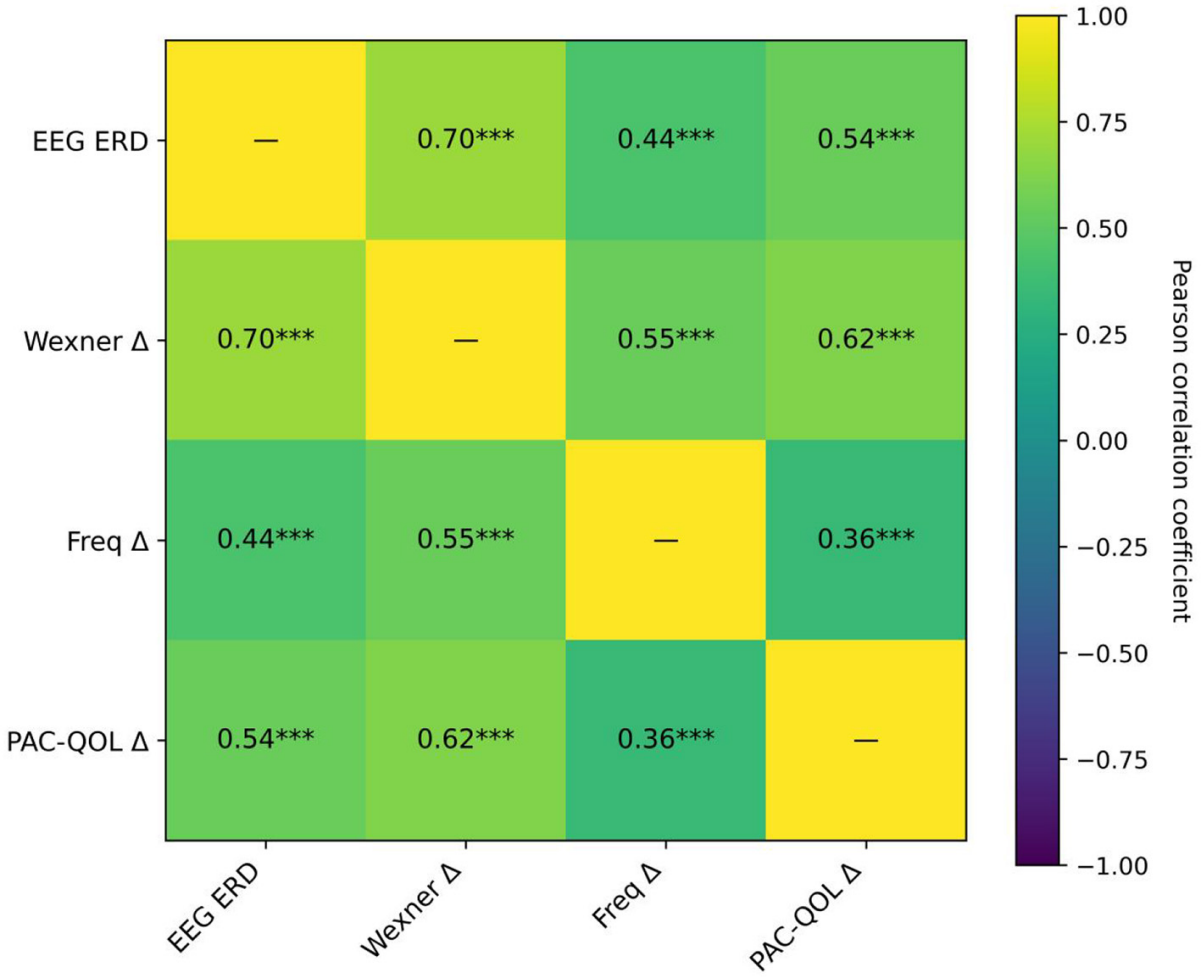
Correlation between EEG ERD changes and clinical improvements in the overall cohort. The symbol ***indicates statistical significance (*P* < 0.001).

As illustrated in Figure 3, correlation analysis in the overall cohort revealed that EEG ERD changes were positively correlated with improvements in Wexner constipation scores, weekly bowel movement frequency, and PAC-QOL scores. Greater ERD enhancement was associated with larger Wexner score improvement (*r* = 0.70, *P* < 0.001), greater bowel frequency improvement (*r* = 0.44, *P* < 0.001), and greater PAC-QOL improvement (*r* = 0.54, *P* < 0.001). These findings suggest that cortical excitability changes captured by EEG were associated with the degree of clinical recovery. The corresponding scatter plots for these correlations are provided in Supplementary Figure 1.

## Discussion

The present retrospective analysis suggests that a multi-modal rehabilitation package—mirror therapy coupled with defecation-focused motor imagery and adjunctive rTMS—yields clinically meaningful improvements in bowel function among patients with frontal lobe infarction. Compared with routine care, the intervention group exhibited larger reductions in Wexner Constipation Scores, more frequent spontaneous bowel movements, and better PAC-QOL outcomes after eight weeks. At the neurophysiological level, μ/β-band ERD increased during defecation imagery in the intervention group, and the magnitude of ERD change correlated moderately with Wexner score improvement (r≈0.56) and weak-to-moderately with bowel-frequency gains. Taken together, these findings point to a convergent signal: cortical engagement indexed by ERD coincides with symptomatic relief, and combined sensorimotor and neuromodulatory strategies appear to facilitate that engagement. However, given the retrospective and correlational nature of the study, the observed associations between ERD enhancement and clinical improvement should not be interpreted as evidence of direct causality.

The ERD signature has long been understood as a reduction in synchronized oscillatory power reflecting cortical activation during movement execution and imagery, with μ (8–13 Hz) and β (≈14–25 Hz) rhythms dominating the sensorimotor profile (21, 22). Links between ERD magnitude and corticospinal excitability—as captured by motor-evoked potentials and intracortical inhibition—support ERD's mechanistic relevance to plasticity processes that rehabilitation aims to harness (23). Work in motor-imagery paradigms and BCI settings further indicates that modulating μ/β-ERD is trainable and behaviorally meaningful, strengthening the case for ERD as a treatment-sensitive readout (14). In stroke cohorts, evolving reviews highlight ERD and related EEG metrics as promising markers that track recovery trajectories and may stratify responders in neurorehabilitation trials (13, 15). Against that backdrop, our observation that greater ERD change accompanies greater bowel improvement is consistent with ERD's role as an index of task-specific cortical recruitment and raises the possibility that EEG could serve not only as a mechanistic window but also as a practical biomarker for bowel-focused rehabilitation.

The plausibility of a cortical mechanism for defecation control is supported by neuroimaging of pelvic-floor and anorectal tasks. Voluntary pelvic-floor contraction robustly engages the supplementary motor area (SMA) and superomedial primary motor cortex, alongside insular and cingulate nodes, indicating that intentional control of continence and expulsion recruits canonical motor-control circuitry (4, 5). Classic and contemporary fMRI studies of ano-rectal sensation and rectal distension implicate prefrontal, anterior cingulate, insula, and sensorimotor regions—structures with well-established roles in autonomic regulation and interoception (6, 7, 24). This distributed network aligns well with our protocol that pairs visually enriched sensorimotor practice (mirror therapy), internal simulation (defecation motor imagery), and excitability-oriented neuromodulation (rTMS) aimed at medial frontal territories. In principle, such coupling should reinforce task-contingent recruitment of SMA-mPFC-M1 loops that contribute to coordinated pelvic-floor relaxation and propulsive effort during defecation. Although fMRI would provide more precise spatial information regarding cortical–subcortical visceral networks, it was not feasible in the present study for several reasons. First, this study was based on retrospective clinical rehabilitation data, and fMRI was not routinely performed during the management of post-stroke constipation in our center. Second, repeated fMRI during defecation motor imagery would have imposed additional cost, time, and physical burden on older stroke patients, many of whom had limited tolerance for prolonged scanning. Third, defecation motor imagery performed in the scanner may differ from imagery practice in a real rehabilitation environment. In contrast, EEG is portable, repeatable, and compatible with task-based rehabilitation assessment. Therefore, μ/β ERD was used as an indirect but clinically feasible marker of cortical engagement. Future studies combining EEG with fMRI, anorectal manometry, or colonic transit testing may provide a more complete characterization of the brain–bowel network.

The neuromodulation component is also congruent with contemporary guidance. Evidence-based consensus indicates that rTMS can augment functional recovery when parameters are tailored to phenotype and safety frameworks are respected (25). Updated IFCN safety recommendations specify dosing ranges, seizure-risk mitigation, and screening procedures that are compatible with our protocol, supporting clinical feasibility in older adults with vascular disease under appropriate monitoring (26). While most high-quality rTMS evidence targets limb motor outcomes, its network-level effects—modulating excitatory–inhibitory balance and connectivity in cortical hubs—render translation to autonomic-visceral targets plausible, especially when stimulation is timed to training that engages the same circuits (here, defecation imagery and mirror-evoked visuomotor coupling).

Our clinical measures are standard and interpretable in constipation research. The Wexner (Cleveland Clinic) Constipation Score offers a 0–30 symptom burden scale sensitive to change across conservative and procedural interventions (27); bowel-movement diaries provide a behavioral anchor relevant to patients and payers; and PAC-QOL adds a validated quality-of-life perspective that often moves in parallel with symptom relief but remains independently meaningful (28). The alignment across these endpoints in our cohort enhances confidence that the observed improvements reflect more than measurement artifact. The EEG results provide a complementary mechanistic layer, with μ/β-ERD changes mapping onto symptomatic change at the individual level—a pattern consistent with an engagement-based account of plasticity wherein repeated imagery plus congruent sensory feedback and facilitated excitability consolidate task-specific cortical representations.

Our findings also intersect with broader concepts of the brain–gut axis. Meta-analytic work and mechanistic reviews emphasize reciprocal signaling between cortical networks, autonomic outputs, immune–endocrine cascades, and the enteric nervous system (29). Stroke can perturb these axes, predisposing to constipation through reduced mobility, medication effects, and disrupted central autonomic control; conversely, persistent constipation has been linked to worse cerebrovascular risk profiles and rehabilitation participation. In that context, data-driven, cortex-engaging rehabilitation may help correct maladaptive central contributions to bowel dysregulation—an interpretation compatible with our ERD–symptom correlations and with imaging literature that highlights prefrontal–cingulate–insular involvement in visceral sensation and motor control.

Several practical implications follow. First, pairing imagery with vivid visual feedback (mirror therapy) appears to be more than motivational—it probably amplifies sensorimotor resonance, boosting the likelihood of measurable ERD changes and behavioral gains. Second, embedding rTMS sessions adjacent to imagery practice may exploit transient windows of increased plasticity, a scheduling principle widely employed in limb rehabilitation and sensible to test in bowel-focused programs. Third, incorporating EEG into routine assessment could help identify responders early, guide parameter tuning (e.g., imagery difficulty, session duration), and standardize mechanistic endpoints for future trials. Finally, although our protocol targeted frontal midline regions, the distributed network observed in pelvic-floor and rectal tasks suggests that individualized targeting (e.g., SMA vs. mid-cingulate vs. insula-adjacent nodes) merits exploration as imaging-guided neuromodulation becomes more accessible. From a clinical perspective, the intervention protocol is relatively feasible to integrate into routine neurorehabilitation programs because mirror therapy and motor imagery are low-cost, non-invasive, and easily standardized techniques. The addition of rTMS may further enhance treatment efficacy in centers with neuromodulation capability, supporting the development of bowel-focused neurorehabilitation pathways in stroke care.

This study has several limitations that should be acknowledged. First, the retrospective, single-center design limits causal inference and increases the risk of selection bias, residual confounding, and unequal distribution of unmeasured clinical characteristics between groups. Because treatment allocation was based partly on physician decision-making and patient preference rather than randomization, patients receiving the combined intervention may have differed in motivation, rehabilitation engagement, or clinical suitability. Although baseline variables were generally balanced and sensitivity analyses yielded consistent findings, the possibility of residual bias cannot be excluded. Second, the absence of a sham rTMS control arm prevents definitive attribution of the observed effects to the neuromodulation component independent of behavioral training. Because mirror therapy, motor imagery, and rTMS were delivered as a combined intervention package, the individual contribution of each component could not be isolated. Future randomized factorial or sham-controlled studies are needed to determine the relative contribution and potential synergistic effects of each modality. The improvements observed may therefore reflect synergistic or non-specific therapeutic influences. Third, bowel frequency was recorded using patient diaries and quality-of-life outcomes were self-reported, both of which are susceptible to reporting bias. Objective physiological assessments—such as colonic transit studies or anorectal manometry—were not included and would strengthen future investigations. Fourth, EEG analyses were based on scalp-level spectral ERD measures during motor imagery. While these metrics are clinically feasible and widely used, source-localized and connectivity-based analyses could provide more precise insights into network-level reorganization. In addition, the present analysis focused mainly on μ/β-band ERD as a task-related cortical activation marker. Other EEG-derived features, such as source-localized activity, functional connectivity, phase synchronization, and frequency-specific motor imagery responses, were not fully analyzed in this retrospective dataset. These aspects should be further investigated in future prospective studies with standardized EEG acquisition and data-sharing protocols. Fifth, potentially influential factors including medication use, dietary intake, fiber and fluid consumption, and physical activity were not fully standardized, and may have moderated treatment effects. Finally, follow-up was limited to the 8-week intervention period, precluding evaluation of long-term durability or optimal maintenance strategies. Moreover, as the cohort consisted exclusively of patients with frontal lobe infarction, generalizability to other stroke subtypes remains uncertain. Prospective, randomized, multi-center studies are warranted to confirm and extend these findings.

In summary, a combined program of mirror therapy, defecation motor imagery, and rTMS was associated with greater improvement in constipation severity, bowel frequency, and disease-specific quality of life than routine care, alongside enhanced μ/β-ERD during defecation imagery and a moderate coupling between ERD change and symptom relief. These preliminary findings suggest that targeted cortical engagement may contribute to bowel-function recovery after stroke. However, given the retrospective and exploratory nature of the study, the clinical utility of EEG-derived ERD as a biomarker remains investigational and requires confirmation in prospective, randomized controlled trials with larger sample sizes.

## Data Availability

The raw data supporting the conclusions of this article will be made available by the authors, without undue reservation.
