# Supplementary material for: Mirror therapy combined with defecation motor imagery and repetitive transcranial magnetic stimulation improves post-stroke constipation: a retrospective study with EEG biomarker analysis
Source: Front Neurol. 2026 Jul 10;17:1818303. doi: 10.3389/fneur.2026.1818303 (PMC13403001; doi:10.3389/fneur.2026.1818303)
Supplement: Supplementary file 1 [file Supplementary_file_1.docx]

Supplementary Figure 1 shows scatter plots of the associations between EEG ERD changes during defecation motor imagery and clinical improvements in the overall cohort. Panels A–C show the correlations between EEG ERD changes and Wexner constipation score improvement, weekly bowel movement frequency improvement, and PAC-QOL score improvement, respectively. Each dot represents an individual patient included in the final analysis. All 98 participants completed the Wexner Constipation Score, weekly bowel movement frequency recording, and PAC-QOL assessment at both baseline and after the 8-week intervention; therefore, no cases were excluded from these correlation analyses because of missing clinical outcome data. Regression lines are shown with corresponding Pearson correlation coefficients and P values. Greater ERD enhancement was associated with larger improvements in constipation severity, bowel movement frequency, and constipation-related quality of life.

**Supplementary Figure 1. Scatter plots showing correlations between EEG ERD changes and clinical improvements in the overall cohort.**

**
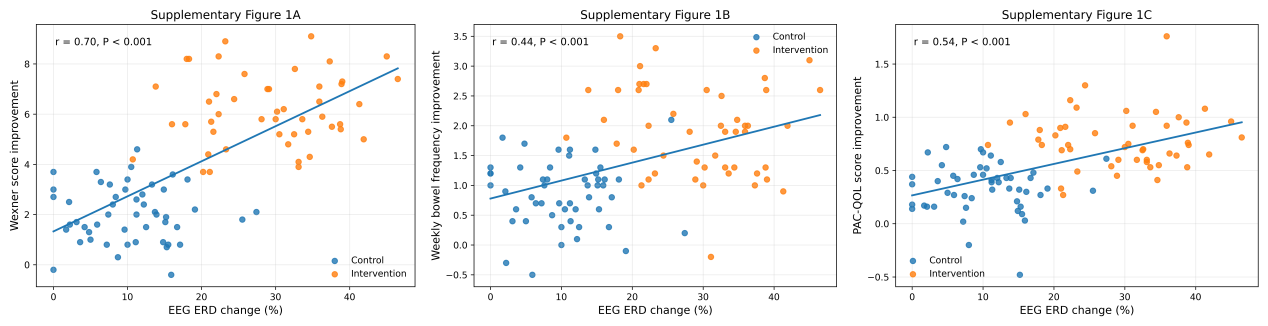
**
